# Adiposity and response to an obesity prevention intervention in Pakistani and Bangladeshi primary school boys and girls: a secondary analysis using the BEACHeS feasibility study

**DOI:** 10.1136/bmjopen-2015-007907

**Published:** 2016-02-09

**Authors:** Geneviève Cezard, Narinder Bansal, Raj Bhopal, Miranda Pallan, Paramjit Gill, Timothy Barrett, Peymane Adab

**Affiliations:** 1 Edinburgh Migration, Ethnicity and Health Research Group (EMEHRG), Centre for Population Health Sciences, University of Edinburgh, Edinburgh, UK; 2 Cardiovascular Epidemiology Unit, Department of Public Health & Primary Care, University of Cambridge, Strangeways Research Laboratory, Cambridge, UK; 3 Unit of Public Health, Epidemiology & Biostatistics, University of Birmingham, Birmingham, UK; 4 Primary Care Clinical Sciences, University of Birmingham, Birmingham, UK; 5 School of Clinical and Experimental Medicine, College of Medical and Dental Sciences, University of Birmingham, Birmingham, UK

**Keywords:** adiposity, intervention, South Asian children, sex, skinfold thickness measurements

## Abstract

**Objectives:**

As a secondary analysis of the BEACHeS study, we hypothesised there would be sex differences in Pakistani and Bangladeshi school children when examining adiposity and their response to an obesity intervention.

**Design:**

The Birmingham healthy Eating and Active lifestyle for CHildren Study (BEACHeS) was designed as a Phase II feasibility study of a complex intervention.

**Setting:**

8 primary schools with predominantly South Asian children in Birmingham, UK

**Participants:**

1090 pupils (aged 5–7 years old) from school year 1 and 2 were allocated at school level to receive an intervention. A total of 574 were enrolled in the study with consent. We focused on the 466 children of Pakistani and Bangladeshi origin (50.6% boys).

**Intervention:**

Delivered between 2007 and 2009, the 1-year obesity prevention intervention targeted school and family-based dietary and physical activities.

**Primary and secondary outcome measures and analysis:**

Adiposity measures including skinfold thickness were compared by sex at baseline and follow-up. Gains in adiposity measures were compared between control and intervention arms in boys and in girls. Measures were compared using two-sample t tests and Wilcoxon-Mann-Whitney rank sum tests according to normality distribution.

**Results:**

At baseline, girls had larger skinfold measures at all sites compared to boys although body mass index (BMI) was similar (eg, median subscapular skinfold 6.6 mm vs 5.7 mm; p<0.001). At follow-up, girls in the intervention group gained less weight and adiposity compared to respective controls (p<0.05 for weight, BMI, waist circumference, central and thigh skinfold) with a median total skinfold gain of 7.0 mm in the control group compared to 0.3 mm in the intervention group.

**Conclusions:**

Our secondary analysis suggests differences in adiposity in Pakistani and Bangladeshi girls and boys and in the effect of the intervention reducing adiposity in girls. These preliminary findings indicate that including sex differences should be examined in future trials.

**Trial registration number:**

ISRCTN51016370; Post-results.

**Strengths and limitations of this study**

- Adding to previous work, we found higher levels of central, peripheral and central-to-peripheral adiposity in Pakistani and Bangladeshi girls compared to boys, despite having a similar body mass index and waist circumference.
- Our secondary analysis shows that a tailored obesity intervention can reduce central and overall adiposity in Pakistani and Bangladeshi girls and emphasises the need to consider sex differences in the development and analysis of future obesity intervention trials.
- Our findings confirm the utility of direct measures of body fat such as skinfold thickness to assess adiposity differences in South Asian children compared to usual adiposity measures that may not detect those differences in specific ethnic and sex groups.
- The BEACHeS study was not designed as a randomised controlled trial but as a feasibility study to assess the feasibility and acceptability of the intervention components and techniques.
- The intervention was a school level intervention and the individual exposure to each component was not collected.

## Introduction

South Asians (SA) have a disproportionately higher burden of diabetes and cardiovascular disease attributed in part to higher levels of central obesity and disproportionate adiposity for a given body mass index (BMI)^1^ compared to their white European counterparts.^2^ ^3^ Fat stored in central compartments is metabolically active and predisposes to dyslipidaemia, metabolic syndrome, diabetes and cardiovascular disease.^4^ Precursors of obesity-associated diseases have also been seen earlier in British-born SA children.^5^ ^6^

The presence and extent of ethnic inequalities in childhood obesity in the UK are hard to determine given the lack of consensus from previous studies,^7^ probably due to the different measures used and the possible inappropriateness of standard BMI cut-offs in SA. Nightingale *et al*^8^ found that British SA children had a higher body fat percentage and higher combined skinfold thickness (mainly due to higher trunk skinfold) despite having lower BMI and a lower waist circumference compared to British white children in England. These findings are consistent with studies using dual energy X-ray absorptiometry (DXA) as a measure of adiposity.^9^ Sex differences in adiposity are also apparent with studies showing a higher percentage of body fat^9^ and skinfold thickness in infant British SA girls^10^ compared to boys. Despite the increased propensity for central obesity and cardiometabolic disease, intervention studies aimed at reducing adiposity in SA children are lacking.

The Birmingham healthy Eating and Active lifestyle for CHildren Study (BEACHeS) developed an obesity prevention programme tailored for children of SA origin at 5–7 years of age, and included a feasibility study to inform a subsequent trial of the intervention. The clinical and cost-effectiveness of the intervention programme is now being evaluated in the West Midlands ActiVe lifestyle and healthy Eating in School children study (WAVES),^11^ a cluster randomised controlled trial which includes 54 schools. With the exception of the ongoing WAVES trial and the developmental DiEt and Active Living (DEAL) study,^12^ ^13^ the BEACHeS study is the only known obesity intervention in SA children in the UK.

We examined several standard anthropometric measures including skinfold thickness to explore adiposity and response to this obesity prevention intervention in Pakistani and Bangladeshi children by sex.

## Materials and methods

### Study population

The methods and main results of the BEACHeS study and intervention are published in detail.^14–16^ Eight Birmingham primary schools with high percentages of SA children were selected. Parents/guardians of each child received a letter distributed through the schools. Our study enrolled 574 school children aged 5–7 years old (school years 1 and 2) with the verbal and written consent of their parents. Baseline data were collected between December 2006 and June 2007 and follow-up data between November 2008 and June 2009. Schools were matched by size and proportion of children eligible for free school meals, and then assigned to either control or intervention status after taking account of the geographical location, in order to minimise contamination between schools. The intervention programme was delivered in four schools over 1 year.

The intervention programme included both dietary and physical activity components, targeting children and their families. These included family cooking workshops and a range of additional physical activity opportunities within and outside of school, all delivered to varying degrees by each school.^16^ A key feature of the intervention development process was the involvement of family, school and community stakeholders, to enable identification of theoretical pathways to behaviour change and relevant barriers and facilitators to achieving these changes. This informed the design and delivery of the intervention programme.^14^ This local stakeholder involvement helped to build an intervention programme that considered the sociocultural and religious context of these communities, and included flexibility in delivery so that intervention components could be adapted by families and schools according to their needs, while delivering the same objectives. For example, while the cooking workshops included the same basic messages around healthy eating, the sample recipes and types of foods discussed varied according to the ethnic mix of those attending. Similarly, schools were provided with a wide range of materials to deliver structured physical activity opportunities during the school day, but were given flexibility in terms of which materials to use and when and how to deliver these.

Child ethnicity was reported by parents and was obtained from the school records. Children were mainly of SA origin (67% Pakistani, 14% Bangladeshi, 5% Indian) with a few from black (8%), white (2%) and other ethnic (4%) backgrounds. We restricted this analysis to Pakistani and Bangladeshi children who were found to be similar in terms of demographic and anthropometric characteristics. Collectively, they formed 81% of the BEACHeS study population.

**Figure 1:**
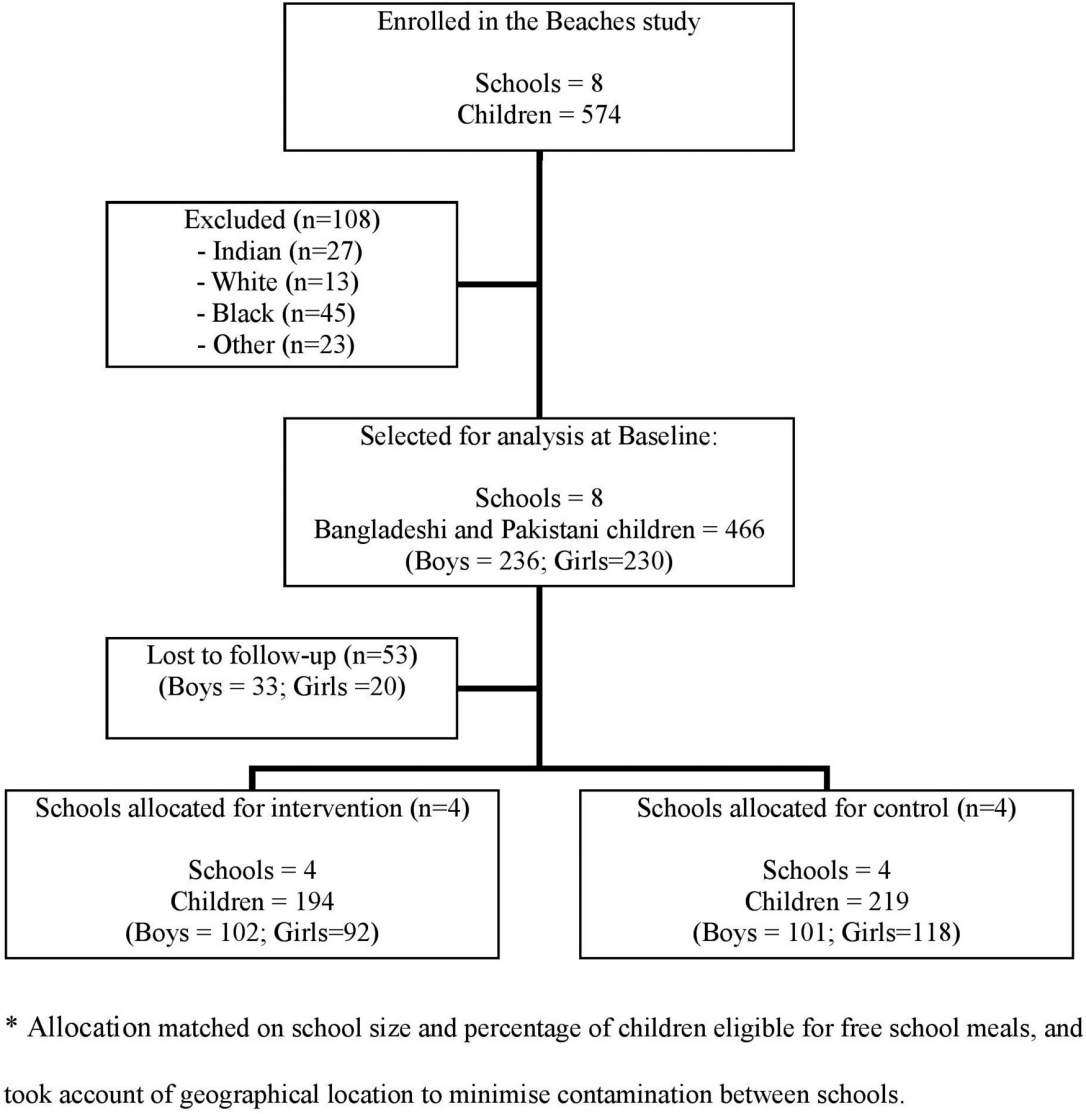
Flow diagram of the cohort selected for analysis. *Allocation matched on school size and percentage of children eligible for free school meals, and took account of geographical location to minimise contamination between schools.

The population analysed (figure 1) comprises 384 Pakistani (82%) and 82 Bangladeshi (18%) children (466 SA in total), of whom 236 (50.6%) were boys. Of these, 413 children (89%) had follow-up information with 101 boys and 118 girls in the control group and 102 boys and 92 girls in the intervention group. Follow-up data was not available for 33 boys and 20 girls, either because the children had left the school, or were absent on the follow-up measurement day.

### Measurements

Equipment for measurement was supplied by Harlow Printing Limited (South Shields, UK). Weight (kg) was taken using a standardised and medically approved Tanita Body Fat Monitor and height (cm) was measured using the Leicester Height Measure. Waist circumference (cm) was measured twice midway between the 10th rib and the iliac crest using a flexible non-stretchable tape measure (Seca 200 BMI body tape) and averaged. Biceps, triceps, subscapular, suprailiac and thigh skinfold thickness measurements (mm) were taken twice using Holtain skin-fold callipers (four times if the difference between the first measurements was greater than 0.4 mm) and averaged for analysis. All measurements were taken by trained researchers following standardised measurement protocols developed for the study (available on request).

BMI was calculated using weight (kg) divided by the squared height (m^2^). BMI SD-scores were derived using the sex-age-specific British 1990 Growth data reference with LMS Growth using Cole's method.^17^ ^18^ The measure of central skinfold was a summation of subscapular and suprailiac skinfold measurements. The measure of peripheral skinfold was a summation of biceps, triceps and thigh skinfold measurements. The measure of total skinfold summed the five skinfold measurements. Central-to-peripheral ratio and waist-to-height ratio (WHtR) were also calculated.

### Data analysis

Data analysis was conducted using SAS V.9.3 (SAS Institute Inc, Cary, North Carolina, USA).

Descriptive statistics on age, ethnicity, language and religion by sex were calculated to show the demographic profile of the population at baseline and follow-up.

For anthropometric measures, mean and SD or median and IQR [q1,q3] were provided depending on the underlying distribution (normality). It is usually appropriate to log-transform non-normally distributed anthropometric measures and use the Edwards transformation for skinfold measures;^19^ however, our data did not reach normality even after log and Edwards transformations. According to normality distribution, two-sample independent t tests or the non-parametric Wilcoxon-Mann-Whitney two-sample rank sum tests were performed to determine sex differences in adiposity at baseline and at follow-up, separately in the control and intervention arms.

Differences between follow-up and baseline measures were calculated. As none were normally distributed, median and IQRs were presented and the non-parametric test was performed to compare changes in adipose measures between the intervention and the control group in boys and in girls. Using a second approach, we analysed the differences in adiposity gain by sex and intervention group and their interaction with linear regressions. The results (not reported) confirmed the observed patterns and are available on request.

**Table 4:** Differences in anthropometric gain from baseline to follow-up between the control and intervention arms in boys and in girls

	Gain in boys					Gain in girls				
	Control		Intervention			Control		Intervention		
Statistics										
Δ Follow-up—Baseline for:	N	Median [q1; q3]	N	Median [q1; q3]	p Value†	N	Median [q1; q3]	N	Median [q1; q3]	p Value†
Weight (kg)	101	5.6 [4.2; 7.6]	101	5.5 [4.0; 7.5]	0.468	117	7.2 [4.2; 9.6]	91	5.0 [3.6; 8.0]	0.008
Height (cm)	101	11.0 [9.8; 12.0]	101	11.2 [9.7; 12.0]	0.640	118	11.5 [9.9; 12.6]	91	10.8 [9.7; 12.0]	0.063
BMI (kg/m^2^)	101	0.8 [0.2; 1.6]	101	0.7 [−0.0; 1.5]	0.348	117	1.1 [0.4; 2.4]	91	0.6 [−0.6; 1.78]	0.005
BMI SD-score (British 1990)	101	0.1 [−0.2; 0.6]	101	0.1 [−0.3; 0.7]	0.627	117	0.3 [−0.1; 0.7]	91	−0.0 [−0.3; 0.3]	0.002
Waist circumference (cm)	101	3.8 [2.3; 6.2]	100	4.0 [1.3; 6.9]	0.909	118	5.3 [2.5; 9.0]	91	3.0 [0.3; 5.8]	<0.001
Waist to height ratio	101	−0.01 [−0.02; 0.01]	100	−0.01 [−0.03; 0.01]	0.802	118	−0.00 [−0.02; 0.03]	91	−0.01 [−0.04; 0.00]	<0.001
Biceps (mm)	100	−0.3 [−1.4; 1.5]	100	−0.5 [−1.4; 0.8]	0.301	115	−0.6 [−2.3; 0.8]	90	−0.7 [−2.2; 0.5]	0.884
Triceps (mm)	100	0.2 [−1.5; 1.8]	100	−0.2 [−1.6; 1.8]	0.999	115	0 [−2.2; 2.4]	89	−0.1 [−1.7; 2.1]	0.786
Subscapular (mm)	100	0.3 [−0.3; 1.9]	98	0.4 [−0.4; 1.6]	0.699	110	1.0 [0; 3.4]	88	0.3 [−0.7; 1.6]	0.009
Suprailiac (mm)	99	0.4 [−0.2; 2.7]	99	0.7 [−0.4; 4.0]	0.483	111	2.2 [0.2; 4.8]	87	1.0 [0; 2.8]	0.036
Thigh (mm)	75	1.8 [−0.6; 5.2]	60	2.2 [0.4; 5.4]	0.441	84	3.0 [0.5; 8.1]	53	1.0 [−0.9; 4.4]	0.011
Total skinfolds (mm)	75	2.9 [−2.1; 11.5]	60	2.8 [−1.3; 12.1]	0.830	82	7.0 [−3.4; 17.9]	53	0.3 [−2.4; 11.5]	0.082
2 Central skinfolds (mm)	99	1.1 [−0.7; 4.6]	98	1.2 [−0.4; 5.3]	0.492	108	3.7 [0.1; 8.3]	86	0.9 [−0.4; 4.0]	0.010
3 Peripheral skinfolds (mm)	75	2.0 [−1.8; 7.7]	60	3.0 [−1.8; 7.7]	0.987	84	3.0 [−2.4; 9.9]	53	−0.1 [−2.5; 6.9]	0.141
Central-to-peripheral ratio	75	0.01 [−0.04; 0.06]	60	0.03 [−0.02; 0.08]	0.238	82	0.06 [−0.00; 0.12]	53	0.04 [−0.00; 0.07]	0.068

†Non-parametric Wilcoxon-Mann-Whitney rank sum test.

BMI, body mass index.

A significance level of 0.05 was used for interpretation.

### Ethics

Informed written consent for study measurements was sought from the parents of all eligible children (those aged 5–7 years in the participating schools), following distribution of detailed information sheets to parents through schools. For those whose parents consented, verbal assent was obtained from children at the time of measurements. If a child did not give verbal assent, the measurements were not taken.

## Results

### Demographic profile

Table 1 shows that boys and girls were similar ages and had the same religion. At baseline, slightly more girls were of Pakistani ethnicity and Urdu as their first language which was reflected at follow-up in the control group but not in the intervention group. The control group had proportionally more Urdu, Bengali and English and less Punjabi and Sylheti as their first language than the intervention group at follow-up. Children were on average 6.5 years old at baseline and 8.4 years old at follow-up.

**Table 1:** Study population's background characteristics

					Follow-up (n=413)			
			Baseline (n=466)		Control (n=219)		Intervention (n=194)	
Variables	Categories	Statistics	Boys	Girls	Boys	Girls	Boys	Girls
		Total N	236	230	101	118	102	92
Age	All	Mean (SD)	6.5 (0.6)	6.4 (0.6)	8.4 (0.6)	8.3 (0.6)	8.4 (0.6)	8.4 (0.6)
		Range: Minimum–Maxium	5–7	5–7	7–9	7–9	7–9	7–9
Ethnicity	Pakistani	N (%)	190 (80.5)	194 (84.3)	77 (76.2)	99 (83.9)	84 (82.4)	75 (81.6)
	Bangladeshi		46 (19.5)	36 (15.7)	24 (24.8)	19 (16.1)	18 (17.6)	17 (18.4)
Age	Pakistani	Mean (SD)	6.5 (0.6)	6.5 (0.6)	8.4 (0.6)	8.3 (0.6)	8.5 (0.7)	8.4 (0.6)
	Bangladeshi	Mean (SD)	6.5 (0.6)	6.4 (0.6)	8.3 (0.7)	8.2 (0.6)	8.3 (0.5)	8.3 (0.7)
Mother tongue	Urdu	N (%)	84 (35.6)	108 (47.0)	40 (39.6)	63 (53.4)	34 (33.3)	32 (34.8)
	Punjabi		37 (15.7)	33 (14.3)	3 (3.0)	6 (5.1)	29 (28.4)	25 (27.2)
	Mirpuri		39 (16.5)	26 (11.3)	17 (16.8)	12 (10.2)	15 (14.7)	11 (12.0)
	Bengali		29 (12.3)	18 (7.8)	25 (24.8)	16 (13.6)	1 (1.0)	2 (2.2)
	Sylheti		18 (7.6)	17 (7.4)	0	2 (1.7)	17 (16.7)	15 (16.3)
	English		4 (1.7)	6 (2.6)	4 (4.0)	5 (4.2)	0	1 (1.1)
	Other		25 (10.6)	22 (9.6)	12 (11.9)	14 (11.9)	6 (5.9)	6 (6.5)
Religion	Muslim	N (%)	125 (98.4)	149 (98.7)	61 (96.8)	77 (98.7)	54 (100)	58 (100)
	Other		2 (1.6)	2 (1.3)	2 (3.2)	1 (1.3)	0	0
	Missing		109	79	38	40	48	34

### Baseline anthropometric measures

Table 2 compares baseline measures in boys and girls. Weight, height, BMI, waist circumference and WHtR were very similar between SA boys and girls. The median BMI was 15.4 kg/m^2^ in girls and 15.3 kg/m^2^ in boys. BMI SD-scores were also similar and close to the 1990 British standard for both SA boys and SA girls, with a mean SD-score close to 0 showing the same BMI level age-sex specific to the British standard. Biceps, triceps, subscapular, suprailiac and thigh skinfold measurements were higher in SA girls at baseline (median total skinfold of 47.1 mm in girls and 38.4 mm in boys; p value<0.001), however, central-to-peripheral skinfold ratios were similar.

**Table 2:** Differences in anthropometric measures between boys and girls at baseline

	Boys		Girls		
Statistics		Mean (SD)*		Mean (SD)*	
Variable	N	Median [q1; q3]	N	Median [q1; q3]	p Value†
Weight (kg)	235	22.1 [19.8; 25.0]	228	22.1 [19.4; 24.7]	0.837
Height (cm)	235	120.0 (6.3)*	229	119.5 (6.0)*	0.334
BMI (kg/m^2^)	235	15.3 [14.3; 16.6]	228	15.4 [14.3; 17.0]	0.785
BMI SD-score (British 1990)	235	0.01 (1.5)*	228	0.03 (1.3)*	0.879
Waist circumference (cm)	234	53.7 [51.1; 57.2]	229	54.1 [50.7; 57.9]	0.632
Waist to height ratio	234	0.45 [0.42; 0.47]	229	0.46 [0.43; 0.49]	0.199
Biceps (mm)	232	6.1 [4.7; 8.4]	225	7.2 [5.7; 9.7]	<0.001
Triceps (mm)	232	9.4 [7.6; 12.0]	225	11.1 [9.3; 14.2]	<0.001
Subscapular (mm)	230	5.7 [4.8; 7.4]	224	6.6 [5.6; 8.9]	<0.001
Suprailiac (mm)	231	5.0 [4.0; 6.8]	224	6.5 [5.0; 9.6]	<0.001
Thigh (mm)	172	12.4 [10.0; 15.8]	179	15.4 [12.2; 19.1]	<0.001
Total skinfolds (mm)	172	38.4 [31.6; 48.4]	178	47.1 [37.6; 60.4]	<0.001
2 Central skinfolds (mm)	230	10.6 [8.9; 14.2]	223	13.1 [10.8; 18.6]	<0.001
3 Peripheral skinfolds (mm)	172	27.5 [22.9; 35.4]	178	34.1 [27.4; 42.3]	<0.001
Central-to-peripheral ratio	172	0.40 [0.35; 0.46]	178	0.41 [0.36; 0.47]	0.257

*Mean and Standard Deviation (SD) are reported for variables that are normally distributed. †Two-sample independent t test or non-parametric Wilcoxon-Mann-Whitney rank sum test depending on underlying normality.

BMI, body mass index.

### Follow-up anthropometric measures

Table 3 shows that weight, height, BMI, waist circumference and waist to height ratio were similar in SA boys and girls, for both control and intervention groups. In the control group, BMI SD-scores were higher at follow-up compared to baseline in boys and girls. In the intervention group, BMI SD-scores had increased in boys but not in girls, with the mean value for girls staying close to the 1990 British standard (mean BMI SD-score (SD)=0.04 (1.5)). At follow-up, skinfold sex differences remained in the control group, that is, girls had higher skinfold measures, whereas in the intervention group sex differences attenuated greatly, especially for the peripheral skinfolds, biceps and thigh skinfolds. Sex differences in central-to-peripheral skinfold ratio increased from baseline in the control group due to a larger increase in girls but remained in the same range in the intervention group.

**Table 3:** Differences in anthropometric measures between boys and girls at follow-up (separately in control and intervention arms)

	Control					Intervention				
	Boys		Girls			Boys		Girls		
Statistics		Mean (SD)*		Mean (SD)*			Mean (SD)*		Mean (SD)*	
Variable	N	Median [q1; q3]	N	Median [q1; q3]	p Value†	N	Median [q1; q3]	N	Median [q1; q3]	p Value†
Weight (kg)	101	27.2 [24.9; 31.4]	118	28.9 [24.4; 34.2]	0.380	102	27.2 [24.5; 32.2]	92	27.3 [22.6; 32.5]	0.552
Height (cm)	101	130.2 (6.5)*	118	130.3 (6.3)*	0.871	102	131.6 (6.4)*	92	130.3 (7.2)*	0.197
BMI (kg/m2)	101	16.5 [14.7; 18.0]	118	17.0 [15.2; 19.8]	0.249	102	15.9 [14.5; 18.0]	92	16.1 [14.3; 18.8]	0.984
BMI SD-score (British 1990)	101	0.34 (1.6)*	118	0.40 (1.4)*	0.756	102	0.14 (1.5)*	92	0.04 (1.5)*	0.642
Waist circumference (cm)	101	57 [53.6; 63.3]	118	60.0 [54; 66.7]	0.136	102	57.1 [53.8; 63.8]	92	55.7 [52.8; 64.2]	0.205
Waist to height ratio	101	0.44 [0.42; 0.48]	118	0.46 [0.43; 0.51]	0.058	102	0.43 [0.41; 0.48]	92	0.43 [0.40; 0.48]	0.604
Biceps (mm)	101	6.0 [4.5; 9.5]	118	7.3 [5.2; 9.6]	0.048	102	5.6 [4.2; 8.8]	91	6.4 [4.5; 9.1]	0.253
Triceps (mm)	101	9.9 [7.2; 14]	118	11.7 [9.6; 15.3]	0.007	102	9.8 [6.5; 13.2]	90	10.9 [9; 14.6]	0.008
Subscapular (mm)	101	6.4 [5; 8.3]	113	8.6 [5.8; 12.3]	<0.001	102	6 [4.9; 8.1]	89	7.1 [2.6; 10.3]	0.007
Suprailiac (mm)	100	6.2 [4.1; 9.7]	113	10 [6.4; 14.34]	<0.001	102	5.9 [4.1; 11.6]	88	7.1 [5.1; 13.2]	0.022
Thigh (mm)	88	14.7 [11.1; 20.0]	92	19.9 [14; 26.7]	<0.001	86	15.2 [11.2; 20.1]	74	15.8 [12.6; 22.4]	0.201
Total skinfolds (mm)	88	42.1 [31.7; 59.3]	90	52.0 [41.1; 76.6]	<0.001	86	43.5 [31.3; 59.8]	74	47.7 [36.3; 61.8]	0.145
2 Central skinfolds (mm)	100	12.3 [9.1; 17.7]	110	18.2 [12.8; 25.7]	<0.001	102	12.1 [9.2; 20.8]	87	14.5 [10.6; 22.4]	0.014
3 Peripheral skinfolds (mm)	88	29.6 [22.8; 41.3]	92	37.4 [29.9; 50.6]	<0.001	86	31.6 [22.2; 42.3]	74	33.0 [25.7; 42.1]	0.183
Central-to-peripheral ratio	88	0.41 [0.36; 0.46]	90	0.48 [0.38; 0.56]	0.008	86	0.42 [0.37; 0.51]	74	0.44 [0.38; 0.51]	0.467

†Two-sample independent t test or non-parametric Wilcoxon-Mann-Whitney rank sum test depending on underlying normality.

BMI, body mass index.

### Changes in anthropometry in follow-up group

Overall, SA boys and girls had a median gain of 6 kg in weight and 11 cm in height between baseline (5–7 years old) and follow-up (7–9 years of age) and about 1 kg/m^2^ of BMI. There was a median gain of 4 cm of waist circumference with no change in the WHtR. There was an overall reduction in biceps skinfold, no change in triceps skinfold and a median gain of 0.5 mm for subscapular, 1.1 mm for suprailiac and 2.2 mm for thigh skinfolds.

Table 4 shows differences in the anthropometric gain from baseline to the end of follow-up between the intervention and control groups, in boys and in girls. In boys, no significant difference in anthropometric gain between the intervention and control groups was found. Whereas girls in the intervention group gained significantly less weight, BMI, BMI z-score, waist circumference, waist to height ratio as well as total central skinfold (subscapular and suprailiac) and thigh skinfold compared to controls.

## Discussion

### Principal findings

The BEACHeS study is the only obesity prevention programme focusing on British SA school children in the UK. This secondary analysis suggests that a tailored obesity prevention programme can reduce central and overall adiposity in British Pakistani and Bangladeshi schoolgirls and highlights the need for inclusion of measures of fat and fat distribution in addition to BMI to detect body composition differences.

### Findings in relation to the literature

Using UK National BMI percentile classification, Balakrishnan *et al*^20^ found that SA boys were more likely to be overweight or obese than girls at 5–7 years of age. However, our findings are consistent with previous work demonstrating the inability of BMI to reveal subgroup differences in adiposity. Shaw *et al*^9^ showed ethnic and gender differences in percentage body fat measured by DXA in British school children as young as 5 years of age. SA children had the highest proportion of body fat (>25%) and girls in each ethnic group had a higher proportion than boys which BMI did not detect. Similarly, Bansal *et al*^10^ showed that SA girls had more adiposity at birth than boys with higher skinfold thickness. In a UK study, Nightingale *et al*^8^ found higher adiposity measured by sum of skinfolds and bioimpedence in SA children aged 9–10 compared to white Europeans, but lower BMI in SA. In 7–10-year-old British children, Henderson *et al*^21^ found subscapular skinfold thickness to be higher in British Pakistani children compared to their white counterpart as well as higher triceps skinfold thickness after controlling for BMI but there was no difference in BMI. This study, together with previous research evidence, suggests that measures of skinfold thickness have more value than BMI when evaluating adiposity in SA children and may be more discriminating in terms of detecting ethnic and sex differences in response to intervention.

To the best of our knowledge, there are no similar intervention studies focusing on South Asian children. However, several childhood obesity prevention studies have reported sex differences in response to intervention. A meta-analysis of school-based interventions to reduce BMI found a significant BMI reduction in girls but not in boys.^22^ Gortmaker *et al*^23^ reported a positive effect of a curricular intervention targeting diet and physical activity on obesity prevalence in girls but not boys. Similarly, Mo-Suwan *et al*^24^ reported an exercise intervention had the effect of preventing BMI gain in girls but not boys. Lazaar *et al*^25^ reported similar findings, showing a greater effect of a physical activity intervention targeting children aged 6–10 years on skinfold thickness and waist circumference in girls compared to boys. In contrast to these studies, Sallis *et al*^26^ found a gender difference in response to a combined dietary and physical activity intervention in the opposite direction and suggested that girls may need an effective intervention combining physical activity opportunities, health promotion and education. While several studies have considered differential intervention effects by sex, most obesity prevention studies do not consider subgroup effects,^27^ which may lead to missed opportunities for tailoring interventions during implementation. The effect of the BEACHeS intervention on girls but not boys fits with previous findings and may be due to the adherence of girls to specific components. This finding will be explored in the ongoing WAVES trial.

### Limitations of this study

The analysis of sex differences in adiposity and response to the BEACHeS intervention was a secondary analysis. The BEACHeS study was a phase II feasibility study designed to assess the feasibility and acceptability of the intervention components and techniques. As such, this was not a randomised trial and did not aim to detect statistical differences in end outcome between intervention and control arms. Furthermore, as allocation to intervention and control arms was not random, imbalance between groups may have biased the results. We tried to minimise this through matching of schools prior to allocation. Individual exposure to each intervention component was not assessed. Our findings are context specific and our sample may not be representative of other SA populations. There was a lack of statistical power to analyse sex differences in adiposity in other ethnic groups.

This study assessed adiposity differences using BMI, waist circumference and skinfold measures, the latter being specifically useful to look at body fat distribution and central fat in SAs. However, an exact body fat percentage was not measured. DXA has been shown to be a precise measurement but concerns have also been raised about its validity as a gold standard to measure body fat composition.^28^ DXA is also expensive, logistically difficult with children recruited through schools (outside of the health service) and potentially seen as invasive and harmful (due to radiation) by parents, despite the fact that the radiation exposure from a DXA is less than 1/10th that of a chest X-ray.

## Conclusions

Our study suggests that a tailored obesity intervention can reduce central and overall adiposity in British Pakistani and Bangladeshi schoolgirls and highlights the need for inclusion of measures of fat and fat distribution in addition to BMI in the assessment of adiposity in these groups. This emphasises the need for both the development of culturally acceptable components of an obesity prevention programme and the use of appropriate measures of fat composition. Our findings need corroboration and further research is required to identify which intervention component may lead to differential effect in SA boys and girls adiposity levels. As a result of our analysis, the on-going WAVES trial^11^ will now include analysis by sex to provide further evidence.
